# Femtosecond Laser-Assisted Ophthalmic Surgery: From Laser Fundamentals to Clinical Applications

**DOI:** 10.3390/mi13101653

**Published:** 2022-09-30

**Authors:** Quan Yan, Bing Han, Zhuo-Chen Ma

**Affiliations:** 1 Department of Ophthalmology, Shanghai General Hospital, Shanghai Jiao Tong University School of Medicine, Shanghai 200080, China; 2 National Clinical Research Center for Eye Diseases, Shanghai 200080, China; 3 Shanghai Engineering Center for Visual Science and Photomedicine, Shanghai 200080, China; 4 Institute of Medical Robotics, Shanghai Jiao Tong University, Shanghai 200240, China

**Keywords:** femtosecond laser, eye, refractive surgery, corneal surgery, cataract surgery

## Abstract

Femtosecond laser (FSL) technology has created an evolution in ophthalmic surgery in the last few decades. With the advantage of high precision, accuracy, and safety, FSLs have helped surgeons overcome surgical limits in refractive surgery, corneal surgery, and cataract surgery. They also open new avenues in ophthalmic areas that are not yet explored. This review focuses on the fundamentals of FSLs, the advantages in interaction between FSLs and tissues, and typical clinical applications of FSLs in ophthalmology. With the rapid progress that has been made in the state of the art research on FSL technologies, their applications in ophthalmic surgery may soon undergo a booming development.

## 1. Introduction

Femtosecond lasers (FSLs) are a technological breakthrough in ophthalmic surgery over the past few years. The first ophthalmic surgical FSL system was developed for corneal surgery by Juhasz and Kurtz in the U.S. in the early 1990s [1,2]. It has offered ophthalmic surgeons a novel tool for bladeless corneal incisions with high precision. There are theoretical advantages to refractive surgery and corneal surgery over manual techniques in which cornea tissue will be cut [3]. FSL technology has also been further introduced to cataract surgery [4].

Thus far, FSLs have shown great potential for applications in ophthalmic surgery due to prominent advantages, such as high accuracy, high safety, appropriate predictability, and minimally invasive wounds. FSLs can improve the visual quality of patients after surgery. Moreover, FSLs can accurately cut any position in eye tissues, thus having a relatively small impact on other tissues. With rapidly increasing requirements for the safety of ophthalmic surgery, and also the requirement for better visual quality, the application of FSLs in ophthalmic surgery is becoming more and more important.

**Figure 1:**
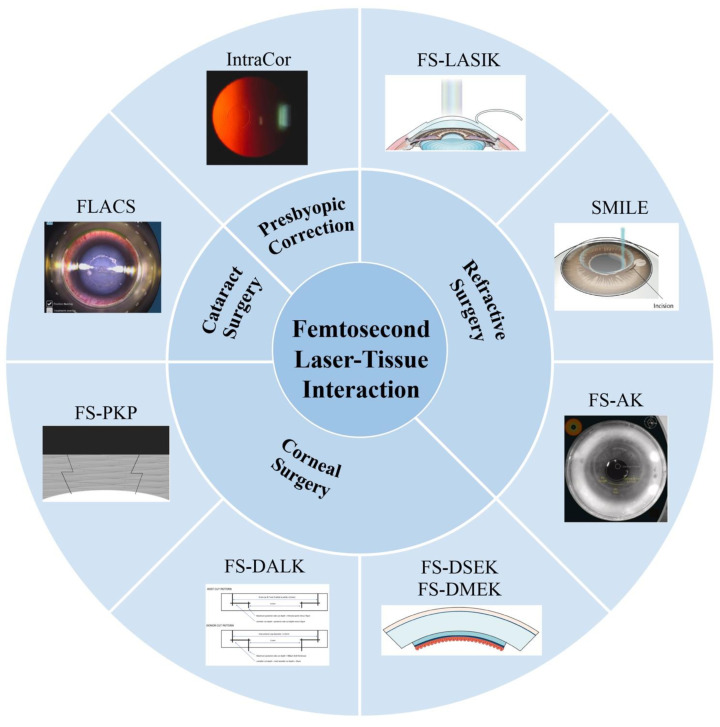
Femtosecond laser-assisted ophthalmic surgery. FS-LASIK: femtosecond laser-assisted laser in situ keratomileusis. SMILE: small incision lenticule extraction. FS-AK: femtosecond laser assisted astigmatic keratotomy. FS-PKP: femtosecond laser-assisted penetrating keratoplasty. FS-DALK: femtosecond laser-assisted deep anterior lamellar keratoplasty. FS-DSEK: femtosecond laser-assisted Descemet’s stripping endothelial keratoplasty. FS-DMEK: femtosecond laser-assisted Descemet’s membrane endothelial keratoplasty. FLACS: femtosecond laser-assisted cataract surgery. Reproduced with permission from authors [5,6,7,8,9,10,11].

In this review, we summarize the recent progress in FSL-assisted ophthalmic surgery. As shown in Figure 1, the fundamentals of FSLs and the clinical applications have been comprehensively reviewed. The advantages of FSLs with regard to these advantages are discussed, which have contributed greatly to the development of clinical applications. Typical FSL-assisted ophthalmic surgery, including refractive surgery, corneal surgery, and cataract surgery, are reviewed. Finally, the current challenges and future research directions are discussed.

## 2. Femtosecond Laser Technology

### 2.1. Fundamentals of Femtosecond Lasers

FSLs feature the merit of a short duration and operate in a manner of ultrafast pulses [12,13,14]. FSLs are thousands of times faster than the pulse produced via other electronic methods, which is usually in the range of tens of to hundreds of femtoseconds [15]. With a very high instantaneous power, FSLs can be focused in a very small space volume [16]. Their energy intensity can be stronger than that of sunlight radiation, which can dramatically increase the intensity of electromagnetic fields [17,18].

Laser fabrication is a process based on the interaction between light and matter, in which a small amount of material is removed or modified from the surface [19]. It can be described in two ways. One is called thermal processing, that is, the strong vibration between lattices leads to the denaturation or separation of materials from the surface as a result of heat; the other is called photo-processing, which is directly based on the breaking of a wide range of valence bonds. It is also generally called cold processing. Light has a certain wavelength and frequency. When light irradiates on the surface of an object, electrons absorb photon energy and change kinetic energy and potential energy [20].

Laser microfabrication, especially pulsed laser fabrication, can be applied to true three-dimensional processing due to its high power density and wide range of processing materials [21]. FSLs can easily achieve a spatial resolution of less than 100 nm [22]. When the light beam is tightly focused into a transparent, photosensitive material, the interaction process is dominated by the nonlinear absorption in the focusing volume, and the optical absorption coefficient shows an exponential nonlinear relationship with the light intensity. So, it can obtain a very high processing resolution, so as to promote the development and application of ophthalmic laser surgery.

Due to the above-mentioned unique advantages of FSLs, they have played an increasingly important role in ophthalmic surgery.

### 2.2. Interaction between Femtosecond Lasers and Tissues

FSLs can produce very high peak power localized in a very small focus volume for a very short period of time, which can ionize the tissue. FSLs have the advantages of short pulse width and small damage, so the application of FSLs in the ultra-fine cutting of biological tissue is critical [20]. FSLs can be focused very accurately in 3D and do not damage other undesired tissues. Since the wavelength, power, repetition rate, and spot size can all be quantitatively adjusted, the interaction between FSLs and tissues can be easily tuned [20]. FSL technology has a pulse duration in the order of 10^−15^ s. The extremely short pulse time effectively avoids the thermal effect of lasers and the impact of shock wave on surrounding tissues. At present, FSL technology is mainly used in corneal tissue ablation and corneal diopter correction, so as to achieve the treatment of ametropia patients. Unlike the argon fluoride excimer laser, the FSL applied to tissues (e.g., corneal tissue) has a wavelength of 1053 nm, which is hardly absorbed by transparent corneal tissue, so it can be focused on any layer in the cornea. When the laser energy increases to the threshold value to generate plasma, a photochemical fracture can be induced and the resulting plasma, shock wave, cavitation, and gas (CO_2_ and H_2_O) will be able to cut the tissue [6]. Generally, the energy threshold of the breakdown can be adjusted by adjusting (shortening) the pulse duration, so as to minimize the impact of shock waves on nearby tissues. FSLs may also interact with biological macromolecules, causing damage to the cell structure or affecting the function of cells. Therefore, when applying FSL technology, we must choose reasonable laser parameters, so that FSLs can demonstrate full advantages in clinical applications.

**Figure 2:**
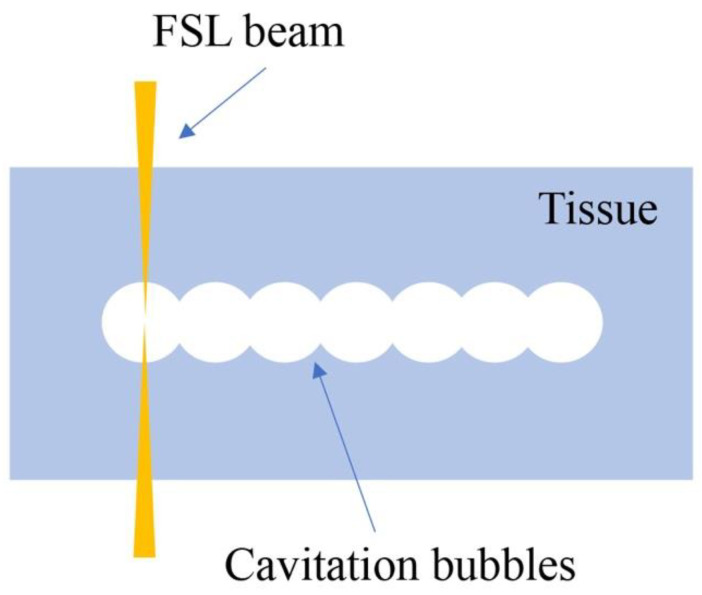
Illustration of photodisruption of FSL in corneal stroma. Cavitation bubbles are developed to cleave the tissue.

Healthy eyes have anatomical structures which are optically transparent, such as the cornea and lens. When using higher power densities of electromagnetic radiation in the near infrared spectrum, such as neodymium-doped yttrium aluminum garnet (Nd:YAG) lasers, these tissues absorb the light energy and then the tissues disrupt, which is called photodisruption (Table 1) [1]. FSLs have an ultra-short pulse duration. When focused in the tissues, FSLs can make small shock waves and develop cavitation bubbles, which can cleave the tissues (Figure 2). Based on this photodisruption mechanism, FSLs are allowed to cut through tissue with micrometer accuracy. The above-mentioned advantages with regard to the inherent ultrafast and high-precision interaction with tissues in 3D make FSL technology a promising tool for ophthalmic surgery [23].

### 2.3. Low-Energy Concept of Femtosecond Lasers

There are two different patterns of photodisruption of FSL. The conventional FSLs use high energy pulses in the range of 4–15 μJ with low frequencies. The cavitation bubbles expand and disrupt the tissue by mechanical forces. Meanwhile, the newer low-energy FSL technology uses high pulse repetition rates (MHz) and low-energy pulses (nJ) [24,25]. The advantages of this new concept of a low pulse energy high repetition rate contains accurate laser focus and decreased stroma gas generation, which make tissue cuts more precise. Unlike the pulses that are at a spot distance from each other in high-energy FSLs, separations are smaller than spot size and they are overlapping in low-energy FSLs. Large numerical apertures in FSL optics make laser spots smaller, which can significantly reduce collateral damage to the surrounding tissue and result in a smooth surface [26,27]. Meanwhile, the cutting speed of FSLs with low pulse energies is similar to that of conventional FSLs because of their high repetition rates.

### 2.4. Ergonomic Aspects of Femtosecond Laser Platforms for Patients

In some commercially available FSL platforms, the patient’s head is placed under a gantry while, in other platforms, a flexible arm with a handpiece is used and can adapt in height and position. FSLs employ two different types of patient interfaces for docking: applanating interface and liquid-filled (non-applanating) interface. In applanating interface, a flat or curved interface touches the cornea directly. This changes the corneal shape but stabilizes the cornea during surgery, which is extremely important in refractive surgery. In liquid-filled interfaces, a vacuum ring touches the sclera and is filled with liquid, which does not change corneal shapes and prevents corneal folds. Thus, these are more suitable for cataract surgery [25,28].

## 3. Clinical Applications

### 3.1. Femtosecond Laser-Assisted Refractive Surgery

#### 3.1.1. Femtosecond Laser-Assisted Laser In Situ Keratomileusis (FS-LASIK)

**Figure 3:**
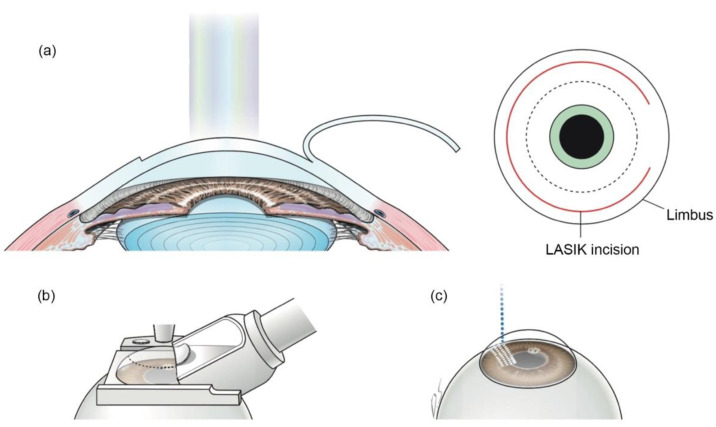
Creation of corneal flap in LASIK and FS-LASIK. (**a**) In LASIK, the corneal flap is created and lifted. (**b**) A mechanical microkeratome is used to make a corneal flap in traditional LASIK. (**c**) In FS-LASIK, small bubbles are created at specific depths in the cornea by FSL. Reproduced with permission from authors [5].

Laser in situ keratomileusis (LASIK) is a surgical correction of refractive errors, especially for myopia patients [29]. In the traditional LASIK procedure, a corneal flap is created by mechanical devices called microkeratomes. Then the corneal flap is lifted, and excimer laser is performed to flatten the corneal stroma layer to change the cornea’s refractive power. When FSL technology is applied, the corneal flap can be made without a blade (Figure 3) [5]. FSLs can create more predictable and reproducible flaps, which have accurate thickness, and epithelial injury can also be decreased [30,31,32]. Meanwhile, FSLs make stromal bridges almost without resistance, thinner flaps, and smoother interfaces compared to excimer laser. These make cornea more mechanically stable [33,34,35]. Furthermore, FS-LASIK can reduce complications such as dry eye, flap hole, and free cap formation [36,37].

#### 3.1.2. Small Incision Lenticule Extraction (SMILE)

**Figure 4:**
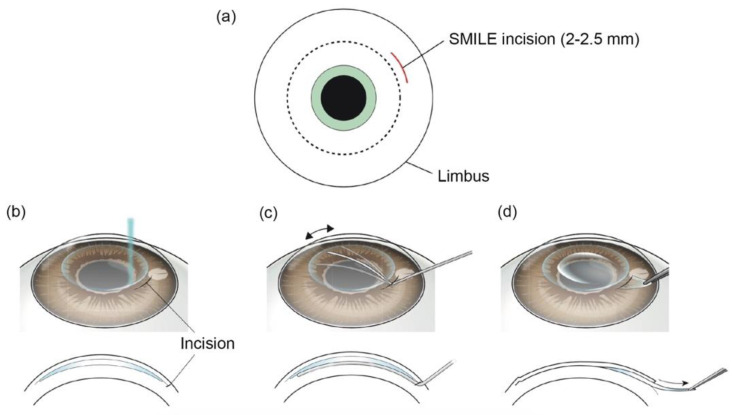
Surgical procedures of SMILE. (**a**) A small corneal incision is made by FSL. (**b**) A lenticule is made by FSL in corneal stroma. (**c**) The lenticule is manually dissected by a spatula through the corneal incision. (**d**) The lenticule is removed through the incision. Reproduced with permission from authors of [5].

SMILE is a kind of refractive surgery to correct myopia and astigmatism. In 2009, SMILE obtained CE approval and was introduced to clinics [38,39]. SMILE creates an intracorneal lenticule using FSLs. The same corneal tissue, which is eliminated by excimer laser in LASIK, is removed in SMILE. A small corneal incision is also made by FSLs at the same time. Then the lenticule is extracted through the corneal incision (Figure 4) [5]. Studies have demonstrated that SMILE has some advantages over LAISK. Because SMILE does not create a corneal flap, many flap-related complications can be avoided, and can improve patients’ intraoperative experience [40,41]. Additionally, dry eye is also decreased after SMILE [42,43]. Studies suggest that SMILE is relatively safe and effective to treat moderate myopia (<–5.0 D) and modest amounts of astigmatism (<–2.0 D), and the visual outcomes are similar to FS-LASIK [44,45]. However, the lenticule made by FSL needs to be manually removed by the surgeon in SMILE. This step is more challenging and highly surgeon-dependent. If the lenticule is not removed successfully, complications may occur and result in poor visual outcomes [46,47]. Further enhancements of platforms, such as nomogram adjustments and eye tracking, might benefit patients undergoing SMILE.

#### 3.1.3. Femtosecond Laser-Assisted Astigmatic Keratotomy (FS-AK)

**Figure 5:**
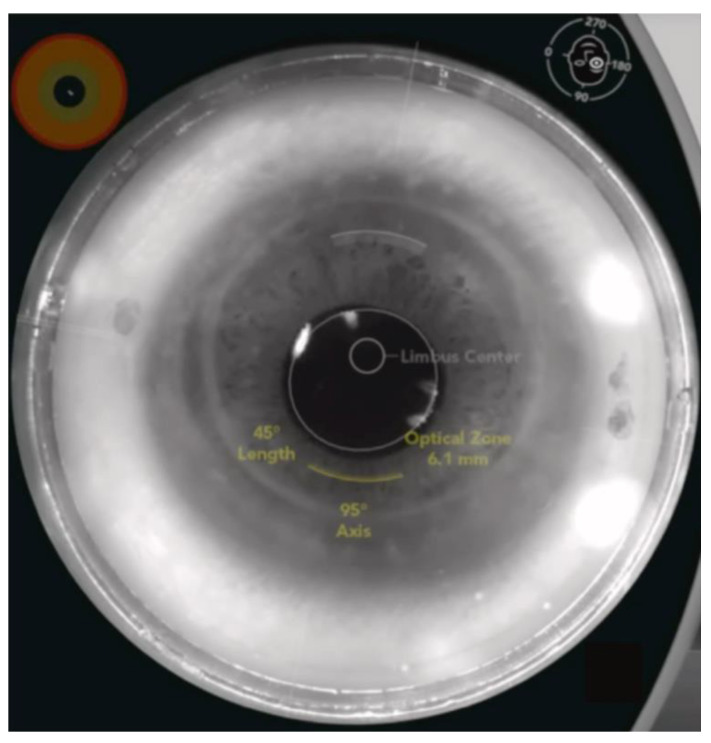
An example of FS-AK using the Catalys FSL platform in an eye after penetrating keratotomy. FS-AK is located on the donor side of the cornea and can be customized in length, depth, location, and treatment axis [6].

Astigmatic keratotomy (AK) has been performed to treat astigmatism for a century. Cutting the cornea with a blade into a specific depth can flatten the corneal curvature in one axis via biomechanical effects. There are three variables in AK surgery: optical zone diameter, AK depth, and arc length. Most AKs’ arc depths range from 75–90% of the corneal thickness at the optical zone. The cuts are performed from the anterior surface of cornea. Intrastromal AKs are performed within the corneal stroma at a depth of 60–90 μm from the corneal surface and 10–20% from the posterior cornea [48]. Traditional AKs are performed manually with a blade. However, manual corneal incision is unpredictable in depth. This may lead to perforation of the cornea or postoperative irregular astigmatism [49]. FSLs have been approved to treat corneal astigmatism with high accuracy. The use of FSLs in AK can make corneal incisions with high precision in length, depth, and shape. All of these advantages could improve patients’ visual outcomes [3,50]. FS-AK also has a lower risk of complications than manual AK. These complications include wound dehiscence, epithelial downgrowth, and infection [6,51]. Generally, the amount of astigmatic correction in native eyes is 0.5 D to 1.5 D [48]. In one patient with naturally occurring high astigmatism, FS-AK was reported to correct astigmatism of almost 6 D in both eyes successfully. In this case, arcuate incisions were carried out at an optical zone of 6.75 mm. Astigmatism was reduced from 5.25 D to 2.75 D in the right eye and from 5.25 D to 2.25 D in the left eye one year after surgery [52]. FS-AK can also treat corneas that are too thin for refractive surgery because of insufficient corneal tissue [53]. Kankariya et al. reported that corneal topographic astigmatism and visual acuity were improved at 3 months after FS-AK in glaucoma patients who had underwent trabeculectomy [54]. Meanwhile, FS-AK improves visual outcomes and is proven to be effective and safe in treating highly astigmatic eyes after penetrating keratoplasty and deep anterior lamellar keratoplasty (Figure 5) [6,51,55,56]. Recently, FSL with low pulse energy was investigated to perform AK in patients with low to moderate corneal astigmatism. One-year results showed that a stable reduction of corneal astigmatism was achieved as well as preservation of corneal optical quality [57].

#### 3.1.4. Complications of FSL-Assisted Refractive Surgery

In general, FSL-assisted refractive surgery is safe and has a low rate of complications [58]. As refractive surgery is aimed to improve life quality by correcting visual acuity, any complications may affect visual outcomes and lead to patient’s unsatisfaction. Halos, glare, residual refractive error, irregular astigmatism, and corneal scarring are common adverse events [59]. In addition, patients often experience dry eye post-surgery because of the damage to the corneal nerve and decreased tear production [60]. In FS-LASIK, flap-related complications such as flap displacement, lamellar keratitis and epithelial undergrowth are common [61,62]. Fortunately, most of these complications can be cured with topical eye drops. SMILE has no corneal flap, thus there are no flap-related complications. However, SMILE may have complications during the surgery due to manual lamellar dissection and smooth lenticule extraction, as discussed above. Eye docking loss during the FSL cut is also an important intraoperative complication in SMILE, resulting in incomplete lenticule cuts, and the incomplete extraction of lenticules, which can lead to irregular astigmatism post-surgery [40]. In FS-AK, the cornea wounds are closed, which can decrease the incidence of infection. However, any corneal incisions have the risk of complications including infection, inflammation, wound gaping, scarring, and unintended full thickness incisions [51,63,64]. In general, infection after FS-AK alone or FS-AK performed in combination with cataract surgery is rare. A few cases of keratitis after FS-AK were reported [63,65,66]. Thus, FS-AK is generally considered to be safe.

### 3.2. Femtosecond Laser-Assisted Corneal Surgery

#### 3.2.1. Femtosecond Laser-Assisted Penetrating Keratoplasty (FS-PKP)

**Figure 6:**
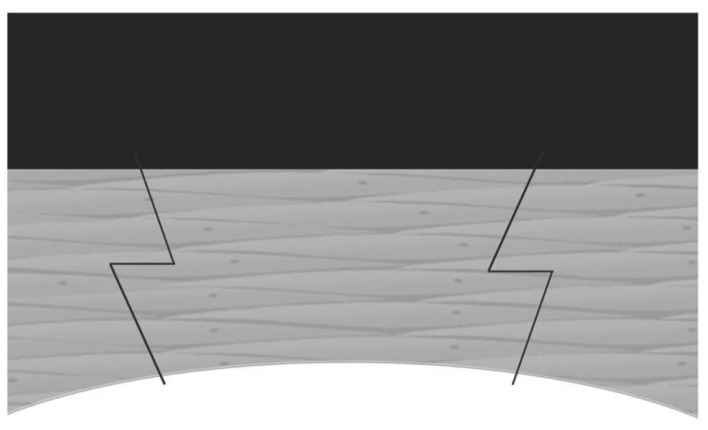
Diagram of FS-PKP with zig-zag incision [7].

Penetrating keratoplasty (PKP), which was first performed more than one hundred years ago, is the mainstay of corneal transplantation surgery to treat various corneal stromal or endothelial diseases. In PKP surgery, the recipient cornea is perpendicularly cut at the center. The donor cornea is cut at the same size and needs to match the recipient cornea. The major complication after PKP is astigmatism. FSLs have a great precision in cutting cornea, and can create advanced-shape corneal cuts such as “zigzag” (Figure 6), “top-hat”, “mushroom”, and “Christmas tree”, which can decrease postoperative astigmatism and improve visual outcomes [7,67]. Farid et al. reported that the patients, who underwent FS-PKP with a zigzag pattern, had a best spectacle-corrected visual acuity greater than 20/30 as well as adequate wound apposition and integrity at 6 months after surgery [68]. Decreased astigmatism in FS-PKP with a mushroom pattern was also reported [69]. FS-PKP also has advances in pediatric patients. The cornea wound made by FSL can be later welded by a diode laser. This can lead to suture-less surgery, which may decrease the need for general anesthesia for postoperative suture management. This can also reduce the risk of endophthalmitis related to suture [70].

#### 3.2.2. Femtosecond Laser-Assisted Deep Anterior Lamellar Keratoplasty (FS-DALK)

**Figure 7:**
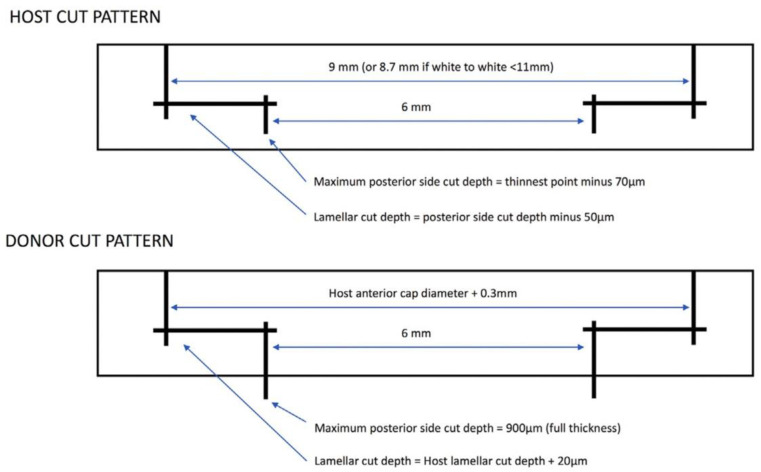
Diagram of cut patterns by FSL in host and donor corneas. The parameters are based on measurements of the host cornea. Reproduced with permission from authors of [8].

DALK is a surgery for corneal stroma diseases, in which the endothelium layer is healthy. DALK selectively removes the central corneal stroma without damaging the underlying Descemet’s membrane. FSLs have the unique ability to create a variety of complex incisions, such as zigzag incision, which are theoretically stronger wound configurations than manual trephination incisions and may result in less astigmatism and earlier visual recovery [71]. FSLs can also customize the parameters of cuts and patterns and have the advantage of making a deep incision close to Descemet’s membrane (Figure 7) [8]. However, FSLs may create irregular interfaces due to the formation of cavitation bubbles in corneal stroma. This may significantly affect the cornea’s optical character [72]. Meanwhile, when the laser cuts are near Descemet’s membrane, they may induce endothelium cells to die [73]. In another study, Li et al. compared the density of endothelial cells in FS-DALK eyes and DALK eyes at 12 months after surgery. They reported there was no significant difference between the two groups. Moreover, epithelial healing was significantly faster in FS-DALK wounds [74]. Therefore, more randomized controlled trials are desired to further access outcomes of FS-DALK compared to manual DALK.

#### 3.2.3. Femtosecond Laser-Assisted Endothelial Keratoplasty

**Figure 8:**
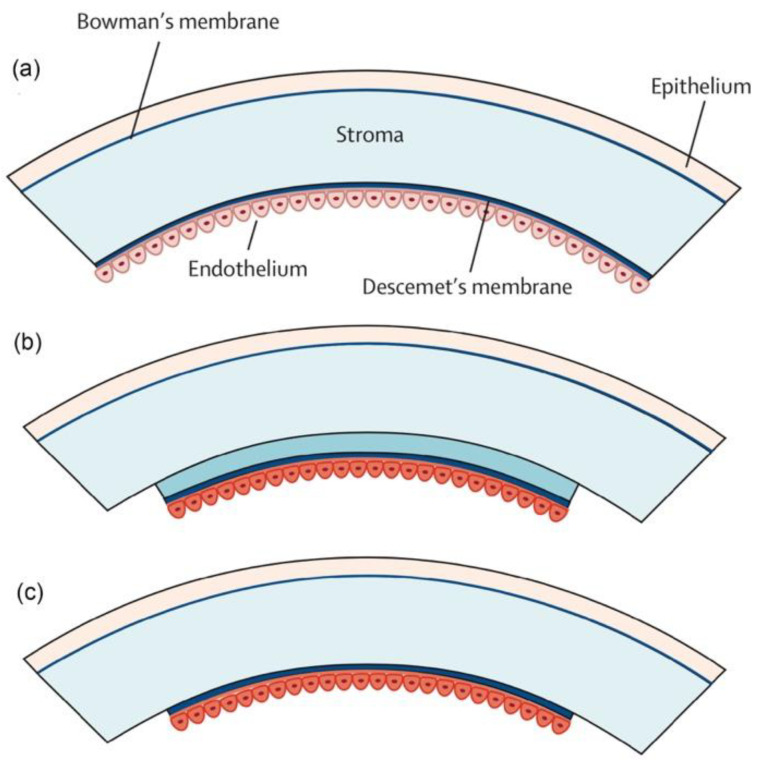
Different types of endothelial keratoplasty. (**a**) The cornea consists of five layers: the superficial epithelial cell layer, Bowman’s membrane, the corneal stromal layer, Descemet’s membrane, and the endothelial cell monolayer. (**b**) Descemet’s stripping endothelial keratoplasty (DSEK). Endothelial cells along with a thin layer of corneal stroma is transplanted. (**c**) Descemet’s membrane endothelial keratoplasty (DMEK). The donor stromal layer is eliminated. Copyright. Reproduced with permission from authors of [11].

Healthy endothelial cells play an important role in maintaining the cornea’s clarity. Endothelial cells are highly specialized and cannot regenerate. In some diseases, such as Fuchs endothelial dystrophy, the endothelial cells are unhealthy or damaged. Descemet’s stripping endothelial keratoplasty (DSEK) is a surgery to remove the nonfunctioning Descemet’s membrane and unhealthy endothelial cells from the recipient cornea. Endothelial cells are then transplanted with a thin layer of corneal stroma (Figure 8) [11,75]. FSLs can remove corneal tissues with perfect centration, shape, and size in DSEK surgery [76]. As discussed above in FS-DALK, the uneven surface created by FSLs in stroma may also be an advantage in DSEK. This could make the donor endothelial disc easier to adhere to recipient cornea [77]. However, Heinzelmann et al. reported that FS-DSEK led to worse visual outcomes than manual microkeratome-assisted DSEK, perhaps because the dissection surface was rougher in FS-DSEK [78]. Descemet’s membrane endothelial keratoplasty (DMEK) removes all stromal layers of the donor cornea. It is more difficult to operate than DSEK (Figure 8) [11] but DMEK provides faster visual rehabilitation than DSEK, and the rejection rate is also decreased in DMEK [79]. FSL technology provides a novel tool to remove corneal stroma precisely in DMEK. Studies in patients with Fuchs endothelial corneal dystrophy or failed penetrating keratoplasty have showed that the rates of detachment, endothelial cell loss, and reoperation are decreased in the FS-DMEK group compared to the manual DMEK group [80,81].

### 3.3. Femtosecond Laser-Assisted Presbyopic Correction

FSL-assisted surgical therapeutics for presbyopia involves corneal inlay implantation and IntraCor surgery. Corneal inlay implantation changes the anterior surface curvature and the refractive index of the cornea to correct presbyopia. It can also treat presbyopia by increasing the depth of focus. The cornea’s anterior surface is not changed in this surgery [82]. Because of FSLs’ advances in the creation of corneal flap and pocket with great accuracy and precision, FSL-assisted corneal inlay implantation has gained more and more attention [83,84]. Baily et al. reported the visual outcomes of patients implanted with the Icolens corneal inlay. The corneal inlay was implanted in a corneal pocket created by FSL. Patients’ uncorrected near-visual acuity and uncorrected distant visual acuity were both increased at 12 months after surgery [85].

**Figure 9:**
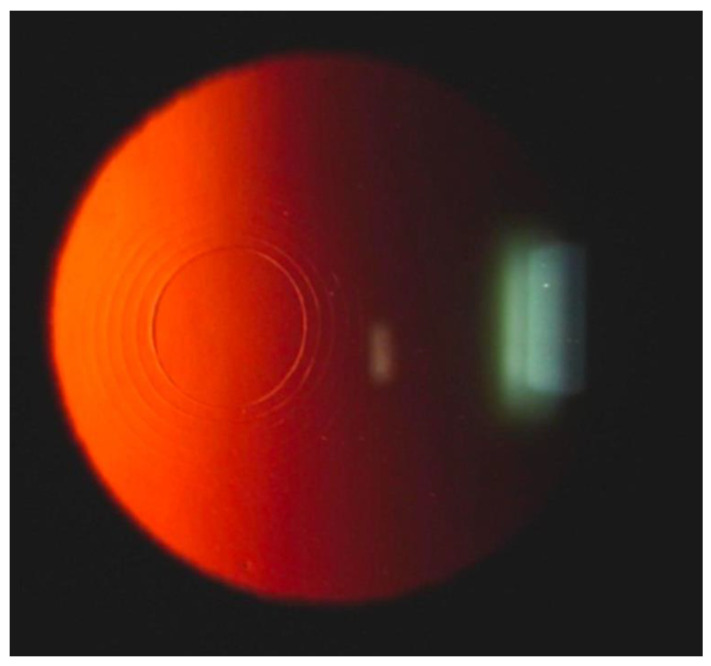
Slit-lamp examination after IntraCor surgery shows concentric cuts in corneal stroma. Reproduced with permission from authors of [9].

IntraCor surgery is a new surgical technique to treat presbyopia. With the help of FSL technology, IntraCor surgery can change the refractive characteristics of the central cornea. Five to eight concentric intrastromal rings are created in the central cornea by FSLs at different depths between the Bowman’s membrane and Descemet’s membrane (Figure 9) [9,86,87]. Holzer et al. investigated early visual outcomes of IntraCor surgery. Patients’ uncorrected near-visual acuity increased significantly while uncorrected distance visual acuity only changed slightly at 3 months after IntraCor surgery [86]. This result shows that IntraCor surgery is promising but still needs further evaluation.

### 3.4. Femtosecond Laser-Assisted Cataract Surgery (FLACS)

**Figure 10:**
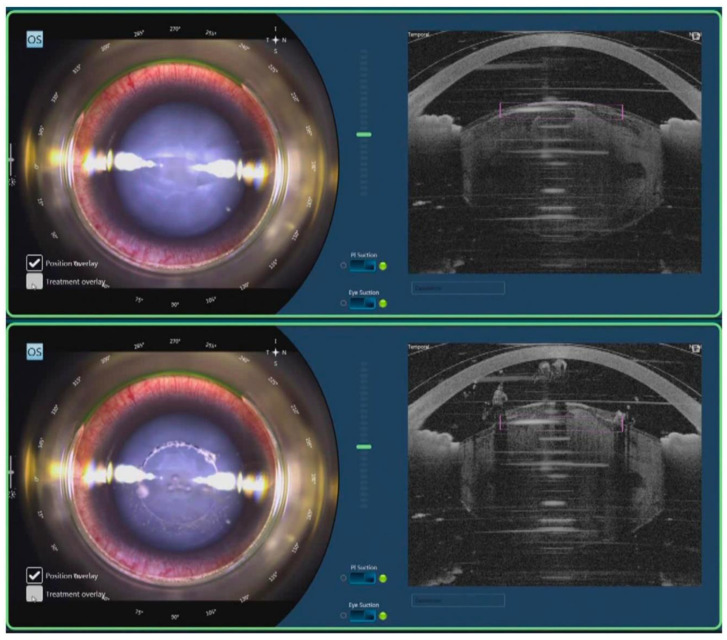
FLACS for white cataract. After docking, real-time OCT shows images of cornea, iris, and lens (**Top**). FSL cuts the anterior lens capsule in a cylindrical pattern in capsulotomy step (**Bottom**). Reproduced with permission from authors of [10].

FSL was first introduced to cataract surgery in the U.S. in 2010. Classic phacoemulsification surgery to treat cataract has been widely used in clinics. Since patients’ visual expectations increased, premium intraocular lens (IOL), such as multifocal and toric IOLs have gained surgeons’ and patients’ attention. Thus, predictability and accuracy of surgery has become more and more important. FLACS offers patients with more accurate and customized surgery than manual phacoemulsification. Additionally, FSL can be very useful in complex cataract cases, such as subluxed cataracts, in which manual capsulorrhexis is difficult [23], and white cataract (Figure 10) [10]. The FSL platform contains the following steps in order: docking, anterior segment imaging, capsulotomy, lens fragmentation, and corneal incisions. The docking process varies between different FSL platforms, including a noncontact apparatus and contact apparatus. After the eye is well-docked, anterior segment optical coherence tomography (OCT) shows real-time images of the cornea, iris, and lens. Anterior and posterior lens capsules can also be clearly imaged. After reviewing the images and checking the treatment parameters, the surgeon starts the laser. Then capsulotomy, lens fragmentation, and corneal incisions are successively performed by FSL.

In the capsulotomy step, FSLs cut the anterior lens capsule in a cylindrical or spiral pattern. The diameter of the capsulotomy is preprogrammed by the surgeon but optimal capsulotomy size is still under discussion [88,89]. It is recommended that the residual anterior lens capsule should cover the optic region of IOLs. This way, the post-operative opacification of posterior lens capsule can be reduced. FSLs can cut the anterior lens capsule in a very centered, round, and regular shape. This advantage provides perfect capsule overlap and IOL stability, which is important for premium IOL implantation [90,91,92,93]. Surgeons can customize the pattern of lens fragmentation on different FSL platforms. For example, surgeons can choose a grid or radial pattern on the Catalys platform, and they can choose a concentric ring or radial pattern on the Victus platform. The lens becomes soft and easy to remove by phacoemulsification after lens fragmentation. The energy amount of following phacoemulsification can also be reduced [91]. However, whether FSL-assisted fragmentation impacts endothelial cell loss is still under investigation. FSLs can create clear corneal incisions and paracentesis with less gaping and leakage than manual incisions [94]. Main incision, biplanar or triplanar, and side port incisions can be made and customized to the desired shape and length. Because the corneal incisions made by FSLs are regular and smooth, complications like Descemet’s membrane detachment and endothelial misalignment are reduced when compared to manual incisions [95]. When treating cataract patients with keratometric astigmatism, arcuate incisions can also be made by the FSLs at the same time of FLACS and can be customized in length, depth, location, and treatment axis [96]. Arcuate incisions in FLACS are similar to FS-AK, which is discussed extensively above in Section 3.1.3. They have been shown to help patients with low astigmatism degrees to achieve excellent anatomic and visual outcomes [97].

Common complications associated with FLACS are anterior capsule tear, incomplete capsulotomy, capsular blockage syndrome, posterior capsule tear, endothelial cell damage, and subconjunctival hemorrhage [3,98,99]. In addition, Darian-Smith et al. reported that pretreatment in FLACS may lead to a higher transient intraocular pressure (IOP) rise and residual IOP after vacuum undocking in glaucomatous eyes than eyes without glaucoma [100]. However, there was a significant decrease in IOP after FLACS. The IOP returned to baseline levels after 1 week and the decrease in IOP persisted through 3 years in glaucoma patients [101]. No significant changes in visual field mean deviation and visual field index were found from the baseline to 12 months after surgery in glaucoma patients [102].

### 3.5. Future Applications of Femtosecond Laser in Ophthalmic Surgery

FSL can be used to change the refractive index on almost all currently used IOL materials after cataract surgery. It is possible to fine-tune the implanted IOL with FSL to eliminate residual refraction errors. The basic configuration of the IOL optics can also be postoperatively changed. This benefit can restructure a multifocal IOL into a monofocal lens and vice versa [103]. Another benefit of FSL in ophthalmic surgery is primary posterior laser capsulotomy. It is possible to perform posterior capsulotomy using FSL at the end of FLACS, which has the potential benefit to prevent posterior capsule opacification. A prospective trial showed the safety of FSL-assisted primary posterior capsulotomy but the long-term effects still need further investigation [104]. Recently, FSL-assisted trabeculotomy was shown to lower IOP significantly in a perfused human anterior segment model. FSLs may potentially provide a noninvasive treatment for primary open angle glaucoma [105].

## 4. Conclusions

In summary, FSL technology has dramatically improved surgical techniques and revolutionized ophthalmic surgery in the past decades. However, we should realize that FSL-assisted ophthalmic surgery contains more steps and overall surgical time may increase. At the same time, FSL technology is still too expensive for a lot of patients. Thus, FSL-assisted ophthalmic surgery should not be overlooked when compared to manual surgery. Apart from this, clinical trials have confirmed the safety and efficacy of FSL technology, especially in long-established LASIK and keratoplasty surgery. Furthermore, new applications of FSL technology are also promising in ophthalmic surgery in the future.

## Figures and Tables

**Table 1:** Lasers in ophthalmology.

Laser	Wavelength	Laser-Tissue Interaction
Carbon dioxide	10,600 nm (far infrared)	Photothermal
Nd:YAG	1064 nm (near infrared)	Photodisruption
Femtosecond	1053 nm (near infrared)	Photodisruption
Krypton	531–647 nm (visible light)	Photochemical (coagulation)
Argon	488–514 nm (visible light)	Photochemical (coagulation)
Excimer	193 nm (far ultraviolet)	Photoablation

Nd:YAG = Neodymium-doped yttrium aluminum garnet.

## Data Availability

Not applicable.
